# AIBP and APOA-I synergistically inhibit intestinal tumor growth and metastasis by promoting cholesterol efflux

**DOI:** 10.1186/s12967-019-1910-7

**Published:** 2019-05-17

**Authors:** Tao Zhang, Qilong Wang, Yeqi Wang, Junping Wang, Yongping Su, Fengchao Wang, Guixue Wang

**Affiliations:** 1 Key Laboratory for Biorheological Science and Technology of Ministry of Education, State and Local Joint Engineering Laboratory for Vascular Implants, Bioengineering College of Chongqing University, Chongqing, China; 2 Institute of Combined Injury, State Key Laboratory of Trauma, Burn and Combined Injury, Third Military Medical University, Chongqing, China

**Keywords:** AIBP, APOA-I, RCT, Colorectal cancer, Cholesterol efflux

## Abstract

**Background:**

The roles played by cholesterol in cancer development and progression represent a popular field in the cancer community. High cholesterol levels are positively correlated with the risk of various types of cancer. APOA-I binding protein (AIBP) promotes the reverse cholesterol transport pathway (RCT) in cooperation with Apolipoprotein A-I (APOA-I) or high-density lipoprotein cholesterol. However, the combined effect of AIBP and APOA-I on intestinal tumor cells is still unclear.

**Methods:**

Immunohistochemistry, western blot and qPCR were performed to investigate the expression of AIBP and APOA-I in intestinal tumor tissues and cell lines. The anti-tumor activity of AIBP and APOA-I was evaluated by overexpression or recombinant protein treatment. Cholesterol efflux and localization of lipid raft-related proteins were analyzed by a cholesterol efflux assay and lipid raft fraction assay, respectively.

**Results:**

Here, we reported that both AIBP expression and APOA-I expression were associated with the degree of malignancy in intestinal tumors. Co-overexpression of AIBP and APOA-I more potently inhibited colon cancer cell-mediated tumor growth and metastasis compared to overexpression of each protein individually. Additionally, the recombinant fusion proteins of AIBP and APOA-I exhibited a significant therapeutic effect on tumor growth in Apc^min/+^ mice as an inherited intestinal tumor model. The synergistic effect of the two proteins inhibited colon cancer cell migration, invasion and tumor-induced angiogenesis by promoting cholesterol efflux, reducing the membrane raft content, and eventually disrupting the proper localization of migration- and invasion-related proteins on the membrane raft. Moreover, cyclosporine A, a cholesterol efflux inhibitor, rescued the inhibitory effect induced by the combination of AIBP and APOA-I.

**Conclusions:**

These results indicate that the combination of APOA-I and AIBP has an obvious anticancer effect on colorectal cancer by promoting cholesterol efflux.

**Electronic supplementary material:**

The online version of this article (10.1186/s12967-019-1910-7) contains supplementary material, which is available to authorized users.

## Background

Cholesterol is essential for maintaining both animal cell membrane architecture and cell signaling [1, 2]. The intestine is one of the main organs for cholesterol absorption and excretion in mammals, and aberrant regulation of cholesterol metabolism has long been linked to the gastrointestinal cancer risk [3–5]. Lipid rafts, as cholesterol-enriched plasma membranes, play an active role in the regulation of cell proliferation, apoptosis, migration and invasion, which are important biological processes involved in cancer initiation, development and progression [6–8]. Thus, many functional responses are probably caused by direct or indirect modulation of the membrane cholesterol content, which may be a potential target for anticancer therapy.

APOA-I, a major protein component of HDL, contributes to the RCT pathway and is considered a potential therapeutic agent for preventing a variety of inflammation-related diseases, including cancer [9, 10]. Clinically, the concentrations of HDL and APOA-I were found to be inversely associated with the risk of colon cancer [11]. Genetic interference with APOA-I levels in vivo exacerbates dextran sulfate sodium (DSS)-induced colitis and colitis-associated carcinogenesis, suggesting that APOA-I plays a protective role in colorectal cancer progression [12]. Recently, AIBP was reported to cooperate with HDL to reduce the lipid raft content of endothelial cells by accelerating cholesterol efflux, leading to restriction of cell migration and angiogenesis in vivo and in vitro [13, 14]. In another study, AIBP promoted APOA-I binding to ATP-binding cassette transporter member 1 (ABCA1) on the cell membranes of macrophages to enhance cholesterol efflux, prevented lipid accumulation and reduced foam cell formation [15]. Early studies reported that treating enterocytes with a polyclonal antibody against AIBP inhibited [^125^I] HDL degradation and binding to cholesterol-loaded cells, suggesting that the synergy of AIBP and APOA-I/HDL in regulating cholesterol metabolism may be a universal phenomenon in mammalian cells [16]. Therefore, we hypothesized that this synergy affects intestinal epithelial tumor development and cancer cells’ biological behavior.

To test the hypothesis, we first evaluated the correlation between AIBP/APOA-I expression and intestinal malignant tissues. Then, we examined the synergistic effect of AIBP and APOA-I on intestinal tumor growth and metastasis, as well as cell proliferation, viability, apoptosis, migration, and invasion and tumor-induced angiogenesis. Finally, we further explored the underlying mechanism involved. This study not only expands our understanding of AIBP and APOA-I functions but also provides some new ideas for the development of novel anticancer strategies targeting cholesterol metabolism.

## Methods

### Cell culture

All cell lines were obtained from the Cell Bank of Chinese Academy of Sciences (Shanghai, China, 2016). LIM1863, HIECs, HISCs and HUVECs were maintained in RPMI 1640 medium (Invitrogen Gibco, USA). Caco2, HEK293, HCT116, SW480, SW620, HT29, 841, LS174T, RKO and LOVO cells were maintained in DMEM (Invitrogen Gibco, USA). All culture media contained 10% FBS (Invitrogen Gibco, USA). All cell lines were authenticated and tested negative for mycoplasma contamination by the providers. And the information of the cell lines are listed in Additional file 2: Table S1.

### Lentiviral vector construction and cell transfection

Lentiviral-based expression vectors containing full-length human AIBP, APOA-I or a negative control (NC) coding DNA sequence (CDS) driven by EF1α were provided by GenePharma (Shanghai, China). Cells were transfected according to the manufacturer’s instructions (GenePharma, China).

### Colony formation and CCK-8 assays

Stably transfected cells were seeded at approximately 300 cells/well in a 6-well plate. Two weeks later, colony formation was analyzed as previously described [17]. Moreover, cells were seeded at approximately 1000 cells/well in a 96-well plate, and CCK-8 assays were performed according to the manufacturer’s protocol (7 Sea Biotech, China).

### Wound healing assay

The stably transfected cells were seeded in fibronectin-coated 6-well plates and cultured to confluence. Cells were serum-starved for 6 h and then incubated at 37 °C in 5% lipoprotein-deficient serum (AngYuBio, China) and DMEM. The wound healing assay was performed as previously described [18].

### Migration and invasion assay

Serum-starved HCT116 cells (2 × 10^5^ cells) were collected from the plate, washed, re-suspended in 5% LPDS/DMEM and added to the transwell (8.0-μm pore size) or another transwell coated with purified fibronectin at (20 µg/ml, Sigma) for migration and invasion assays, respectively. Subsequently, cells were seeded into transwell chambers in the presence or absence of CsA (10 µM, Sigma-Aldrich, USA). After 48 h of incubation, the cells that transmigrated onto the lower surface of the filter were stained with crystal violet and counted under a microscope (Olympus, Japan). Three independent experiments were conducted, and the data are presented as the mean ± SD.

### Mouse xenografts and in vivo studies

The stably transfected cells (5 × 10^6^ cells) were implanted into the flanks of BALB/c male nude mice (Nanjing, China). Because all lentiviral-based cells were marked by GFP, tumor growth was monitored in vivo 21 days after transplantation with a Kodak In-Vivo FX professional imaging system (Connecticut, USA). APOA-I recombinant protein (0.5 mg/kg, Sino Biological) was administered subcutaneously once every 3 days at a site away from the cell implantation location. Tumor size was determined by measuring the tumor length (a) and width (b). Tumor volume (V) was calculated according to the formula V = (ab)^2^/2. Additionally, we generated a recombinant protein containing AIBP fused to the C-terminal of full-length APOA-I and linked by peptides Pro-Gly-Ser-Gly-Ser-Gly, which was designated as R-AIBP + APOA-I. R-AIBP + APOA-I, recombinant APOA-I proteins (Sino Biological) and the APOA-I mimetic D-4F (Ac-DWFKAFYDKVAEKFKEAF-NH2) (OntoRes), which were used to treat the C57BL/6J-Apc^Min/+^ mice (Jackson Laboratory). The mice were treated intravenously with BSA (n = 8), D-4F (n = 6), APOA-I (n = 6) or the AIBP + APOA-I combination (n = 7) (0.5 mg/kg) daily until the first tumors from randomly selected mice were detectable, and the mice were then intraperitoneally injected (10 mg/animal) thrice per week until they reached 20 weeks of age. Statistical analysis of the tumor number and size was performed.

### Liver metastasis assay

For liver metastasis studies, 8- to 9-week-old male BALB/c nude mice were used. The abovementioned stably transfected colon cancer cells were harvested and mixed with 50% Matrigel at a concentration of 2 × 10^5^ cells per ml. Experimental liver metastases were generated by intrasplenic injections of 1 × 10^4^ cells (50 µl of cell suspension). After 28 days, liver metastatic foci and mouse survival were analyzed.

### Cholesterol efflux assay

The stably transfected cells (2 × 10^5^ cells) or HCT116 cells were treated with BSA, APOA-I, AIBP or the AIBP + APOA-I combination for 6 h at 37 °C in 5% LPDS and DMEM or EBM. Then, cholesterol efflux assays were performed using the Cholesterol Efflux Fluorometric assay kit (Biovision, USA) according to the manufacturer’s instructions. The following equation was used to calculate the cholesterol efflux percentage: % Cholesterol efflux = (Fluorescence intensity of the media) / (Fluorescence intensity of the cell lysate + media) × 100.

### ELISA

Cell culture supernatants and mouse sera were collected and assayed using APOA-I (R&D Systems, USA) and AIBP (Aviva Systems Biology, China) ELISA kits according to the manufacturer’s protocol.

### Cell fractionation

The stably transfected cells receiving treatment were plated onto 10-cm dishes at 1 × 10^6^ cells per dish. Cells without any modifications were treated with MβCD (10 mM, Solarbio Life Sciences, China) for 20 min and washed twice with ice-cold PBS; the cytosolic fraction (1 ml) was extracted using the ProteoExtract Subcellular Proteome Extraction kit (Merck, Germany). Triton X-100 soluble materials were extracted with 500 µl of TNE buffer (25 mM Tris-HCl, 0.15 mM NaCl, 5 mM EDTA, and protease inhibitors) containing 1% Triton X-100. Insoluble materials were further extracted with 250 µl of TNE buffer containing 1% SDS. Equal amounts of protein from each fraction were analyzed by western blot.

### Western blotting

Western blot analysis was performed with reference to a standard protocol [19]. The following primary antibodies were used: APOA-I (Bioss, Beijing, China); integrin β1, CAV-1 and VEGFR2 (CST, Danvers, MA, USA); and β-actin and GAPDH (Beyotime, Shanghai, China). Other primary antibodies were purchased from Abcam (Cambridge, MA, USA) using the recommended concentrations in accordance with the manufacturer’s instructions.

### Visualization of lipid rafts with cholera toxin B

The stably transfected cells were plated on glass coverslips and analyzed by Vybrant^®^ Lipid Raft Labeling Kits (Invitrogen, USA) according to the manufacturer’s instructions.

### IHC and immunofluorescence (IF) analysis

For experimental mouse or human intestinal and colorectal tissues, IHC and IF staining was performed as previously described [20, 21]. The study methodologies were approved by the local ethics committee, and the following primary antibodies were used: AIBP (Abcam, ab81907, 1:300 dilution), APOA-I (Bioss, bs-0849R, 1:300 dilution) and CD31 antibody (Abcam, ab28364, 1:300 dilution). The corresponding secondary antibodies were used in accordance with the manufacturer’s instructions.

### Quantitative RT-PCR

Total RNA from cells or tissues was extracted using TRIzol reagent (Invitrogen, USA), and cDNA synthesis was performed using the PrimeScript™ RT reagent Kit (Takara, China). Quantitative RT-PCR was carried out using the Bio-Rad CFX96 Touch system with Real-time PCR Master Mix (SYBR Green). The PCR primers are listed in Additional file 2: Table S2.

### Statistical analysis

Statistical analyses between groups were performed by two-tailed Student’s t-test to determine significance when only 2 groups were compared. Statistical comparisons among 3 or more groups were performed using one-way ANOVA followed by the Tukey test or Dunnett’s test. Differences were considered significant at the level *P* < 0.05 (*P < 0.05, **P < 0.005, ***P < 0.001). The data are expressed as the means ± SDs. Kaplan-Meier curves were used to compare survival times among groups. All statistical analyses was performed using SPSS 16.0.

## Results

### AIBP expression and APOA-I expression are associated with the malignant degree of intestinal tumors

**Fig. 1:**
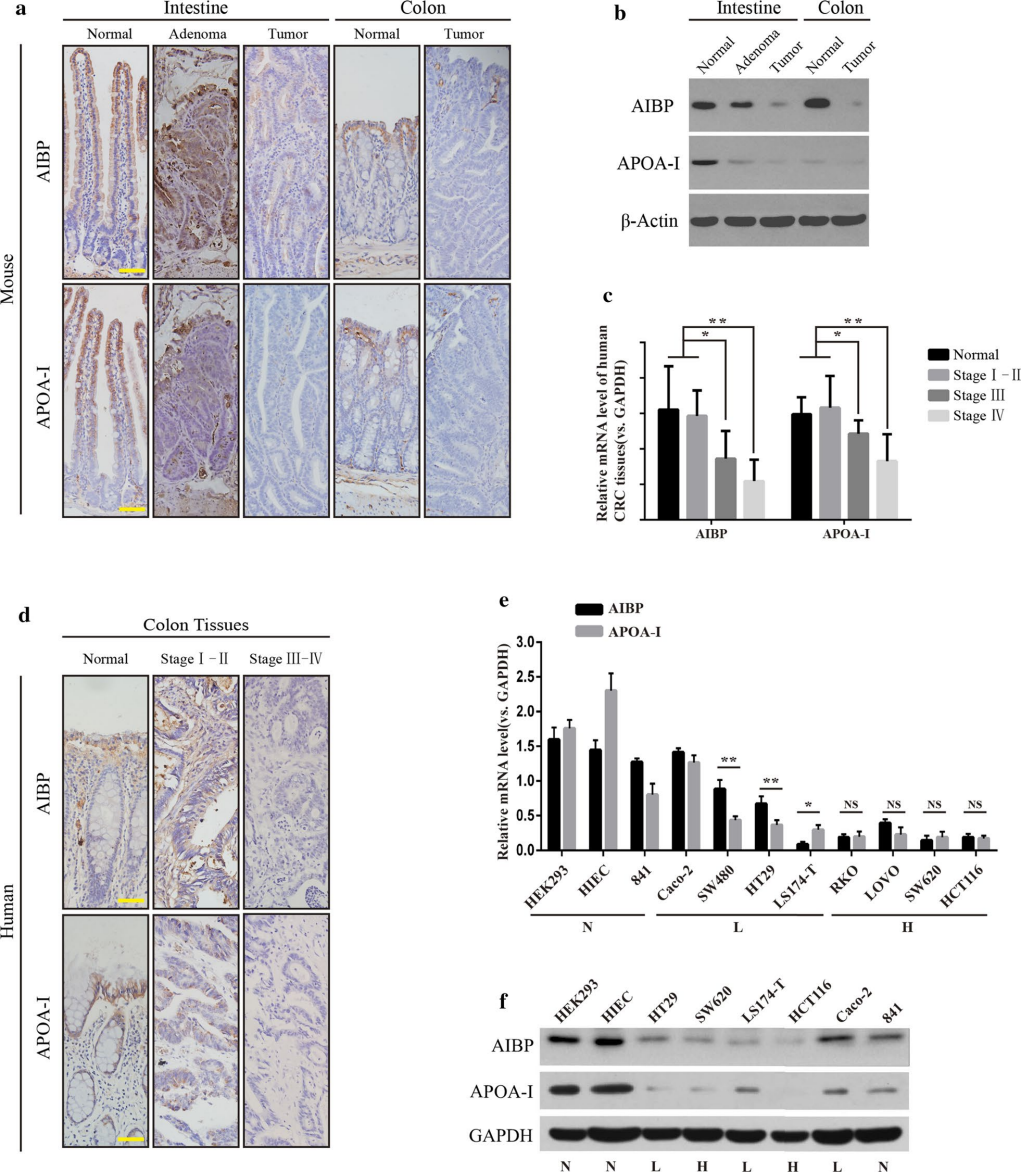
APOA-1 and AIBP expression in normal and neoplastic intestinal tissues. **a** IHC staining for AIBP and APOA-I in normal intestinal tissues, small intestinal adenoma, large tumors and colon tumors in C57BL/6 J-Apc^Min/+^ mice; scale bar, 50 μm. **b** Western blot for APOA-1 and AIBP in intestinal normal, adenoma and tumor tissues in C57BL/6 J-Apc^Min/+^ mice. **c** AIBP or APOA-I mRNA expression in the patients’ normal and cancerous tissues was evaluated according to the tumor differentiation stage (n = 47; *P < 0.05; **P < 0.01; ***P < 0.0001). **d** Staining for AIBP and APOA-I in human normal and malignant colon tissues from patients; scale bar, 50 μm. **e**, **f** qPCR and western blot results showing AIBP or APOA-I mRNA expression in various cell lines, including normal and colon cancer cell lines with a varying degree of malignancy. N, normal epithelial cells; L, low-malignant colon cancer cells; H, high-malignant colon cancer cells (NS, no significant difference; *P < 0.05; **P < 0.01)

APOA-I is synthesized predominantly in the liver and small intestine [9], and AIBP is reportedly ubiquitously expressed in various organs [13], but their expression in malignant conditions of the gastrointestinal tract remains unknown. Here, we examined AIBP or APOA-I expression in normal intestinal epithelial tissues, adenomas (from Apc^min/+^ mice ranging in age from 100–150 days), and large tumors (Apc^min/+^ mice ranging in age from 200 to 230 days) [22, 23]. The IHC and WB results showed that APOA-I expression was obviously decreased in the intestinal adenomas, while AIBP expression was slightly reduced compared with those in the normal tissues. All large tumors, whether in the intestine or colon, exhibited markedly weak staining of both APOA-I and AIBP compared with normal tissues (Fig. 1a, b). Moreover, we analyzed the expression of AIBP/APOA-I in 47 pairs of patients’ cancerous and normal tissues. The results showed that both AIBP and APOA-I levels were significantly downregulated in stage III–IV Colorectal Cancer (CRC) compared to those in normal tissues or early stage I–II CRC (Fig. 1c, d; Additional file 2: Table S3). These results indicated that in the mouse adenomas, only APOA-I expression was significantly downregulated, whereas in the large tumors in mice and human advanced cancer tissues, the expression levels of both were consistently decreased to a minimum.

To further determine the correlation between AIBP/APOA-I and the malignant degree of intestinal tumors, we analyzed their expression in colon cancer cell lines with different malignant degrees. The results showed that the expression patterns of AIBP and APOA-I were inconsistent in cancer cells with low malignancy, such as HT29, SW480, and LS174-T cells, while those in relatively highly malignant cells, such as SW620 and HCT116 cells, were consistently decreased to a minimum (Fig. 1e, f).

Taken together, these results suggested that low expression levels of both AIBP and APOA-I were associated with the malignant degree of intestinal tumors, and such low expression levels may provide a favorable condition for intestinal tumor progression.

### AIBP and APOA-I cooperate to inhibit tumor growth and metastasis

**Fig. 2:**
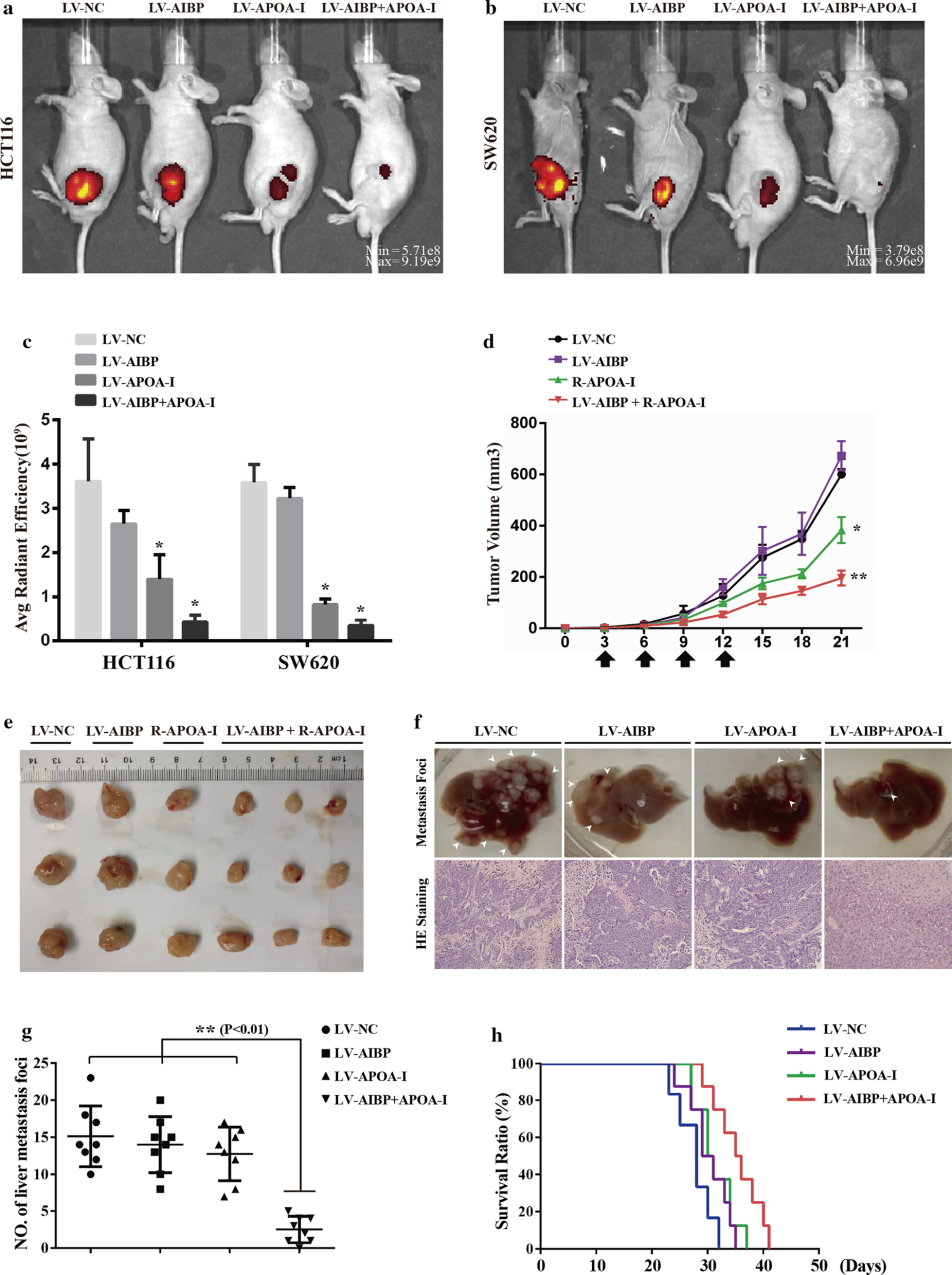
AIBP and APOA-I cooperate to inhibit tumor growth and metastasis. **a**–**c** Representative images and statistical analyses of tumors in mice inoculated with HCT116 (**a**) or SW620 cells (**b**) expressing the negative control (NC), AIBP, APOA-I and AIBP + APOA-I, and the average radiant efficiency [(p/s/cm^2^/sr]/(μW/cm^2^)] of tumors in mice (**c**) (n = 5; *P < 0.05; **P < 0.01). **d** The tumor growth rates were evaluated by calculating the tumor volume. **e** Representative tumors from mice injected with HCT116 cells expressing AIBP and subcutaneously administered APOA-I recombinant proteins (R-APOA-I, 0.5 mg/kg) at the end stage (mean ± SD, n = 5; *P < 0.05; **P < 0.01). **f** Representative picture and HE staining image after injection of the indicated cells. **g** Liver metastatic foci were quantified (mean ± SD, n = 8, **P < 0.01). **h** The survival rates of the indicated cell-transplanted mice (n = 6, 8, 8, and 8, respectively). Kaplan-Meier survival curves representing the percentages of live mice on the days indicated. Statistical significance among the studied groups was calculated using GraphPad Prism 6 software

AIBP and APOA-I have been reported to frequently work in a cooperative manner [24, 25]. The above results showed that simultaneous low expression of both is associated with the malignant degree of intestinal tumors, implying that the concurrent existence of AIBP and APOA-I may inhibit colon cancer cell-mediated tumor growth. To test this speculation, we established stably overexpressed colon cancer cell lines using HCT116 cells, which were designated as HCT116-NC, -AIBP, -APOA-I and -AIBP + APOA-I (Additional file 1: Figure S1). Then, the cells were grafted into the flanks of nude mice by subcutaneous injection. Tumor growth was monitored in vivo after 21 days, and the results showed that the luminescence intensity was substantially weaker in tumors expressing HCT116-AIBP + APOA-I than that in tumors from the other three groups (Fig. 2a). Consistent results were acquired in the SW620-mediated xenograft tumor model (Fig. 2b, c; Additional file 2: Table S4a). To further determine the combined inhibitory effect of AIBP and APOA-I on tumor growth, we subcutaneously injected only HCT116-AIBP cells into the flanks of nude mice, and the APOA-I recombinant protein was then administered intravenously once every 3 days for 4 cycles. The tumors derived from HCT116-AIBP cells accompanied by APOA-I administration were markedly smaller than those derived from HCT116-NC, HCT116-AIBP, or APOA-I administration-alone cells and had reduced growth rates (Fig. 2d, e; Additional file 2: Table S4b and c). In this experiment, APOA-I overexpression or administration alone inhibited tumor growth to a certain extent, but this effect was significantly enhanced by the addition of AIBP.

To further examine the role of AIBP and/or APOA-I in metastasis of colon cancer cells, the above stably transfected HCT116 cells were injected into the spleens of nude mice (Fig. 2f, g). Fewer liver metastatic foci were observed in the mice injected with HCT116-AIBP + APOA-I cells compared to the other three groups. In parallel experiments, the mean survival time of HCT116-APOA-I + AIBP-implanted mice (35.4 ± 4.0) was prolonged by 7.7 days or 4.1 days compared with that of HCT116-NC or HCT116-APOA-I implanted mice, respectively (Fig. 2h; Additional file 2: Table S5).

Taken together, these results suggested that AIBP and APOA-I synergistically played a significant inhibitory role in colon cancer cell-mediated tumor growth and metastasis.

### Evaluation of the therapeutic effect of AIBP + APOA-I in Apc^Min/+^ mice

**Fig. 3:**
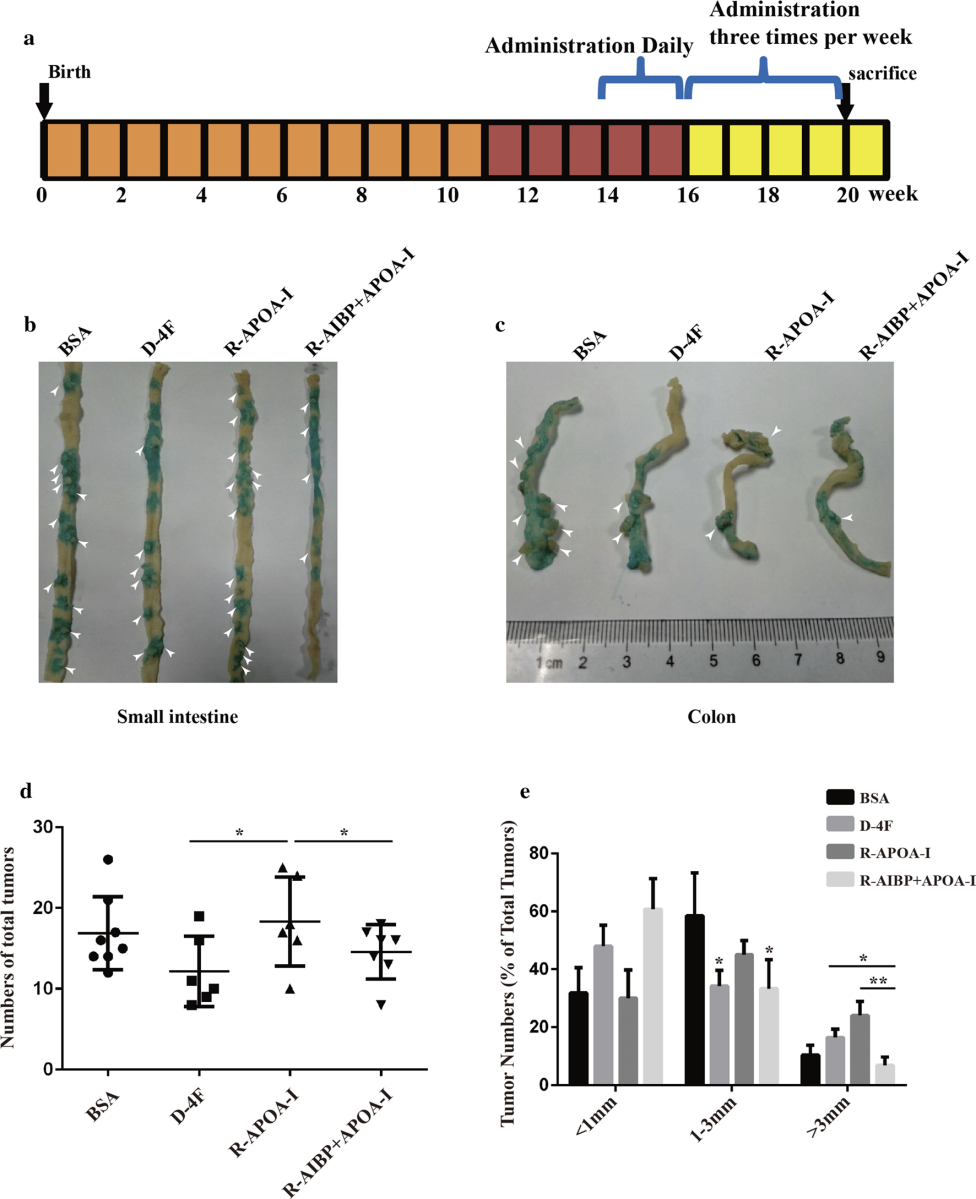
Evaluation of the therapeutic effect of AIBP + APOA-I in Apc^Min/+^ mice. **a** Schematic representation of the recombinant protein treatment protocol as described in the Materials and Methods. **b**, **c** Representative tumor-bearing small intestines and colons were stained with 1% alcian blue, and the white arrows indicate the tumor nodules. **d** The total numbers of small intestinal and colorectal tumors per mouse were enumerated (Mean ± SD, *P < 0.05). **e** The proportion of tumors of different sizes in each group was calculated and analyzed (Mean ± SD, *P < 0.05, **P < 0.01)

To further evaluate the combined therapeutic effect of AIBP and APOA-I on colon cancer intestinal neoplasia in Apc^Min/+^ mice, we generated a recombinant protein containing AIBP fused to the C-terminal of full-length APOA-I and linked by peptides Pro-Gly-Ser-Gly-Ser-Gly, which was designated as R-AIBP + APOA-I. The mice were treated according to the protocol described in Fig. 3a, and APOA-I mimetic peptides (D-4F) were used as positive controls, which have an excellent anti-tumor effect in multiple mouse tumor models [26–28]. To ensure successful administration, serum APOA-I and AIBP levels were monitored by ELISA assays (Additional file 1: Figure S2). The mice were sacrificed on the last day of the experiment, and the intestinal tumors were enumerated immediately. Statistical data showed that compared with BSA treatment, R-APOA-I treatment alone did not significantly affect the numbers of tumor nodules (16.88 ± 1.60 vs. 18.33 ± 2.26), whereas both D-4F and R-AIBP + APOA-I treatment obviously reduced the numbers of tumor nodules (9.50 ± 0.99 and 9.29 ± 1.01, respectively) (Fig. 3b–d). To compare the therapeutic effects of D-4F and R-AIBP + APOA-I, we further calculated the numbers of tumors according to different sizes defined by the diameter of the tumor in mm (< 1 mm, 1–3 mm and > 3 mm). The results showed that compared with BSA or APOA-I, D-4F treatment significantly reduced only the proportion of 1–3-mm tumors, whereas R-AIPB + APOA-I treatment reduced the proportions of both 1–3-mm and > 3-mm tumors (Fig. 3e). These results suggested that AIBP + APOA-I induced an inhibitory effect on intestinal inherited tumors and had some advantages over D-4F in slowing tumor growth and limiting tumor size.

### AIBP and APOA-I in combination inhibited cell migration, cell invasion and tumor-induced angiogenesis

**Fig. 4:**
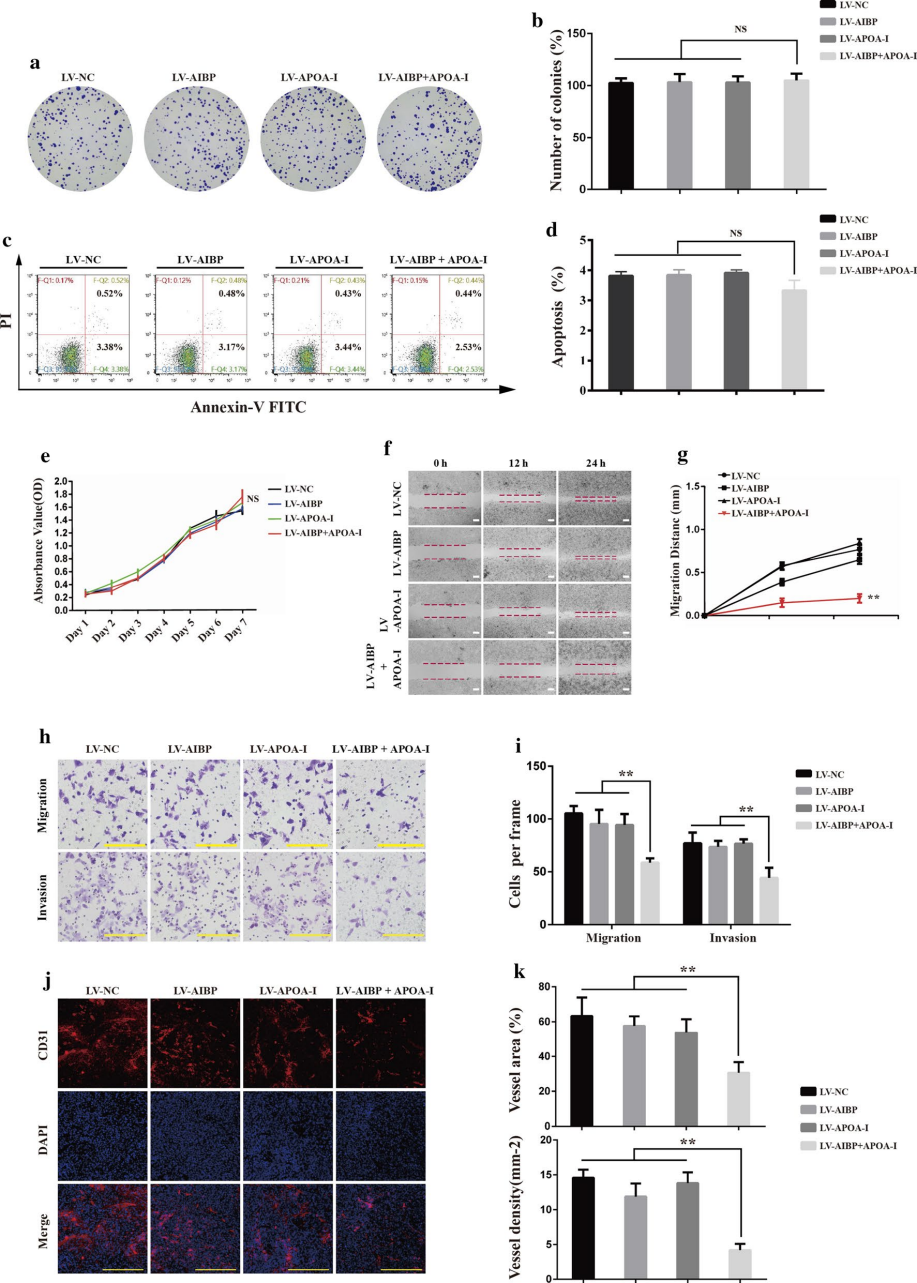
The effect of combined AIBP and APOA-I on colon cancer cells’ biological behavior. **a**, **b** Representative images and statistical analyses of clone formation (mean ± SD, n = 3, NS, no significant difference). **c**, **d** Analysis of cell apoptosis by flow cytometry. **e** The CCK-8 assay for viability of the indicated cells from day 1 to day 7 (mean ± SD, n = 3, NS, no significant difference). **f** Wound healing assay for migration of the indicated cells. Representative images were taken at the indicated time points. Scale bar, 200 μm. **g** The corresponding migratory distance was quantified (n = 3; *P < 0.05; **P < 0.01). **h** Representative fields of the indicated cells passing through the transwell chambers; Scale bar, 100 μm. **i** The number of cells per frame was counted. **j** CD31 staining for tumor-induced angiogenesis. Scale bar, 100 μm. **k** Vessel density (mm^2^) and vessel area (%; mean ± SD, n = 5; **P < 0.01)

To further explore why AIBP combined with APOA-I inhibited tumor growth and metastasis, we examined the effects of AIBP and/or APOA-I on colon cancer cell proliferation, viability, apoptosis, migration and invasion and tumor-induced angiogenesis. We observed that neither individual nor the combined expression of AIBP and APOA-I affected cell proliferation, viability or apoptosis, whereas migration and invasion abilities were significantly inhibited in cells expressing AIBP + APOA-I, but not in cells expressing either protein individually (Fig. 4a–h). Additionally, tumor-induced angiogenesis was evaluated by CD31 antibody staining, and the results showed that the average vessel density and area were lower in tumors derived from HCT116-AIBP + APOA-I cells than those in tumors derived from the other three groups (Fig. 4i, j). These data suggested that AIBP in combination with APOA-I can inhibit tumor-induced angiogenesis, cell migration and invasion, thus inhibiting tumor growth and metastasis.

### The cooperative inhibitory role is dependent on accelerating the cholesterol efflux

**Fig. 5:**
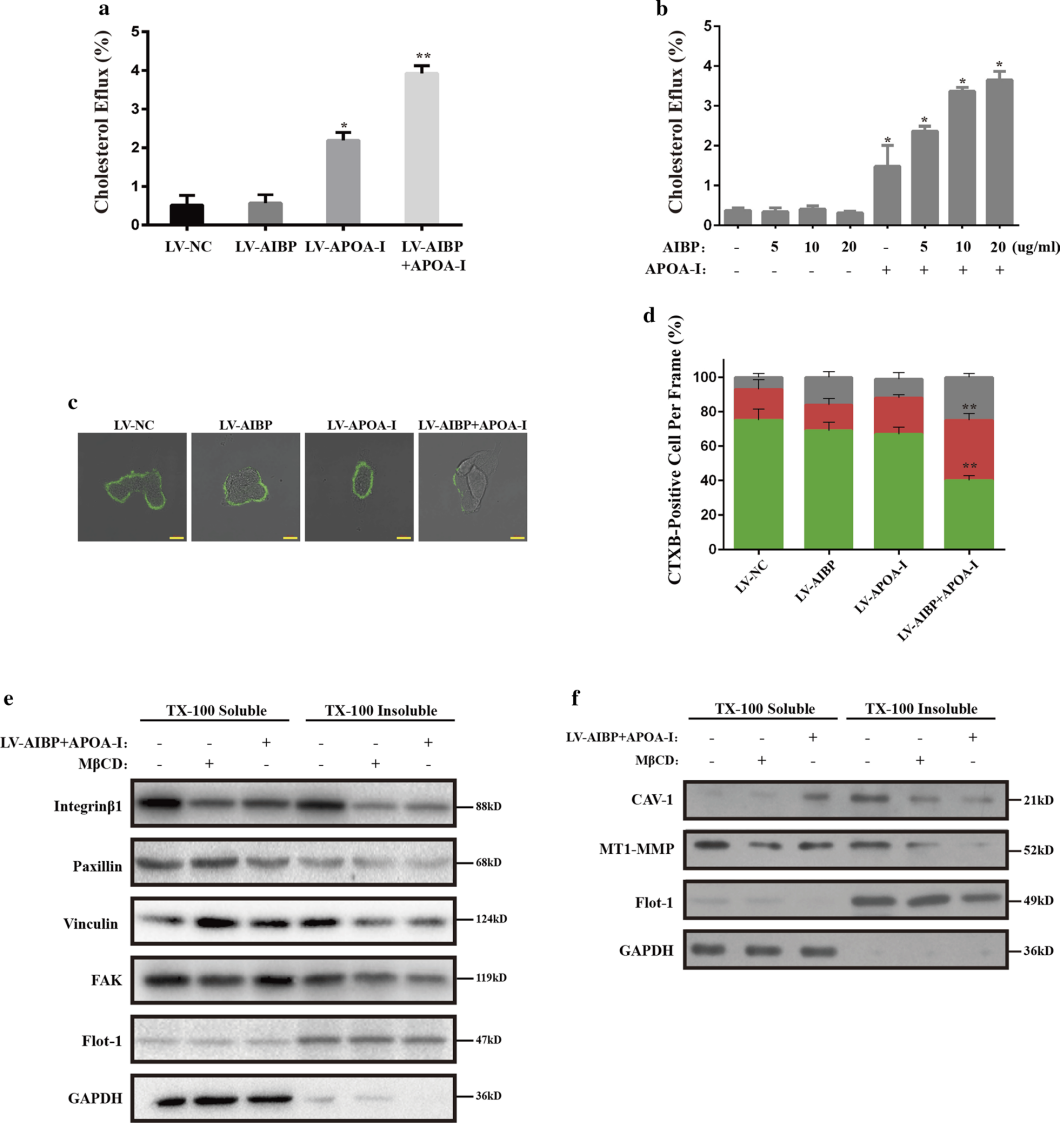
The effect of combined AIBP and APOA-I on cholesterol efflux and raft-related signaling**. a** Cholesterol efflux assay for the indicated cells as described in the Materials and Methods. **b** HCT116 cells were incubated with APOA-I (10 µg/ml) for 6 h in the presence or absence of AIBP (5, 10 and 20 µg/ml) and cholesterol efflux was measured. **c** The indicated cells were stained for lipid rafts with cholera toxin B (CTxB, green). Scale bar, 10 μm. **d** The percentage of cells with high, middle and low lipid raft contents per frame (mean ± SD, n = 10, *P < 0.05; **P < 0.01). **e** The indicated cells with or without AIBP + APOA-I overexpression, or HCT116 cells treated with or without 10 mM MβCD for 30 min; the cell lysates were separated into Triton X-100 soluble and insoluble fractions as described in the Materials and Methods and blotted with integrin β1, paxillin, vinculin, FAK, Flot-1 and GAPDH. **f** The indicated cells were treated the same as in (**f**) and blotted with MT1-MMP, CAV-1, Flot-1 and GAPDH

AIBP in combination with APOA-I reportedly contributes to cholesterol efflux from various types of cells [13, 15, 29]. Thus, we hypothesized that the combined inhibitory effect of AIBP and APOA-I on colon cancer cells is probably achieved through a similar mechanism. To test this hypothesis, we first evaluated the effect of AIBP and/or APOA-I on cholesterol efflux in HCT116 cells. Compared with individual overexpression, AIBP and APOA-I co-overexpression accelerated the cholesterol efflux to a larger extent (Fig. 5a). Meanwhile, the cells were treated with exogenous recombinant protein, and the results showed that APOA-I is required for cholesterol efflux, which was enhanced in an AIBP concentration-dependent manner (Fig. 5b). Accelerating the cholesterol efflux often induces a reduction in the lipid raft content in cells. Then, we evaluated the lipid raft content with CTxB staining. High, middle and low lipid raft contents were defined by the CTB-positive area per cell (high, > 4 μm^2^; middle, 2–4 μm^2^; low, < 2 μm^2^). In the control group, the cells with high, middle and low lipid raft contents accounted for 75%, 18% and 7% of all cells, respectively. No significant changes in these proportions were noted in the individual overexpression groups (APOA-I: high, 69%; middle, 15%; low, 16%; AIBP: high, 67%; middle, 21%; low, 11%). However, in the co-overexpression group, the proportion of cells with a high lipid raft content was reduced to 40%, and the proportions of cells with middle and low lipid raft contents increased to 35% and 25%, respectively (Fig. 5c, d).

The integrity of lipid rafts is necessary for proper localization and functioning of migration- and invasion-related proteins [6]. Localization of integrin β_1_ and FAK [30, 31] or MT1-MMP and CAV1 [32–35] at lipid rafts is closely related to migration and invasion, respectively. We isolated cell raft fractions, and the blot results showed that in the positive control group, MβCD treatment markedly altered the locations of the above proteins on the lipid rafts. Consistent results were obtained in the cells co-overexpressing AIBP and APOA-I, suggesting that the combination of AIBP and APOA-I simulated the effect of MβCD on membrane rafts and interfered with the migration- and invasion-related signaling pathway (Fig. 5e, f).

**Fig. 6:**
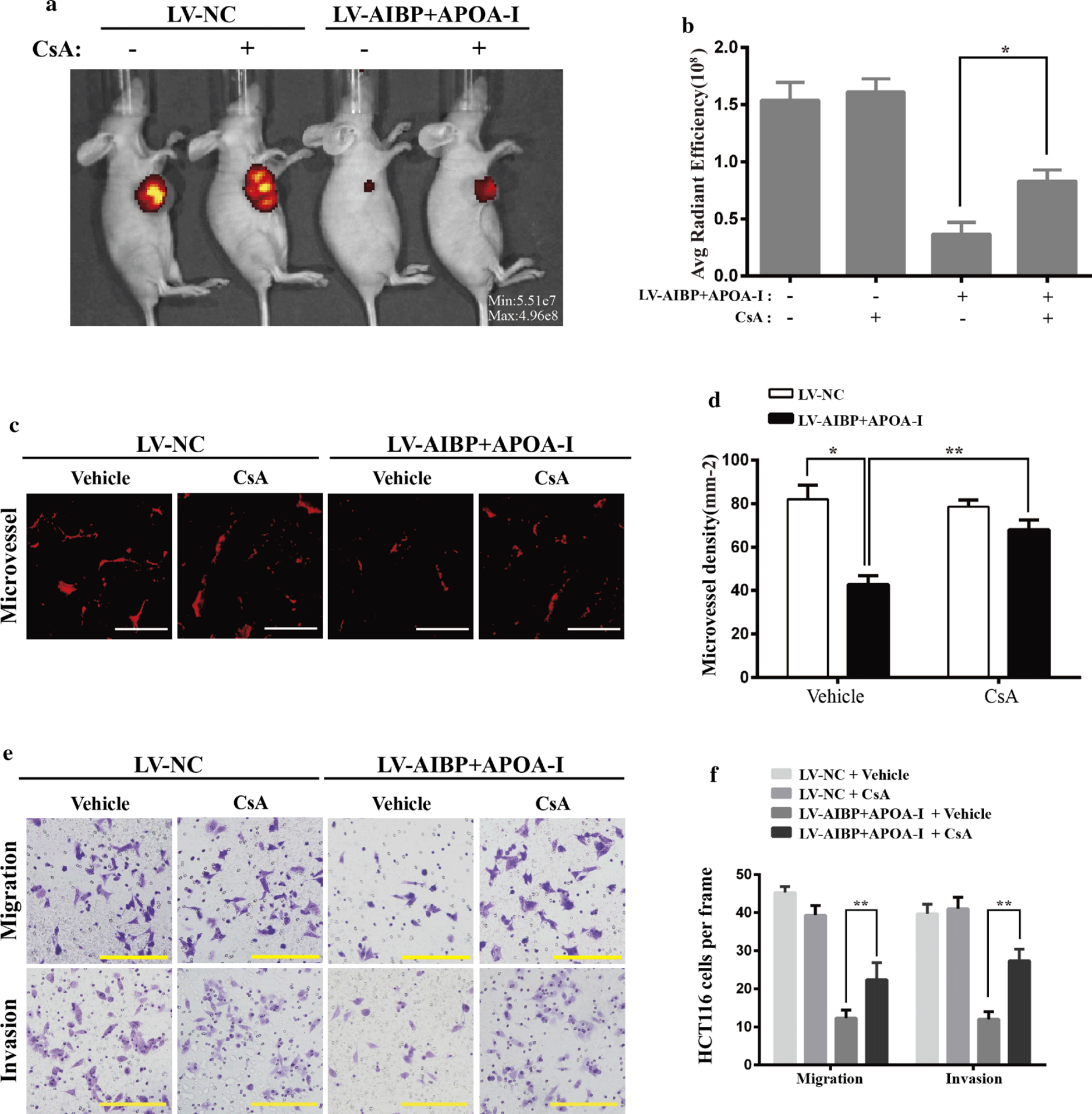
CsA can abolish the combined inhibitory effect of AIBP and APOA-I. **a** Representative images and statistical analyses of tumors in mice inoculated with HCT116 cells co-expressing AIBP and APOA-I upon intraperitoneal administration of CsA (10 mg/kg). **b** The average radiant efficiency [(p/s/cm^2^/sr)/(μW/cm^2^)] of the tumors in mice (mean ± SD, n = 5, *P < 0.05; **P < 0.01). **c** The tumor microvessels were stained for CD31. **d** Vessel density (mm^2^, mean ± SD, n = 5, *P < 0.05; **P < 0.01). **e** Representative images of migration and invasion of the indicated cells treated with or without CsA for 48 h (10 µM). **f** The corresponding cell number per frame was counted. Scale bar, 50 μm (mean ± SD, n = 10; **P < 0.01)

The above results implied that the inhibitory effect of AIBP and APOA-I on tumor growth is probably achieved by promoting cholesterol efflux. To further determine the mechanism, we treated mice inoculated with HCT116-AIBP + APOA-I cells with CsA, which is a cholesterol efflux inhibitor. In vivo imaging results showed that CsA treatment had no significant effect on tumor growth mediated by HCT116-NC but obviously promoted tumor growth mediated by HCT116-AIBP + APOA-I (Fig. 6a, b). Moreover, we found that 10-μM CsA treatment not only had no significant effect on colon cancer cell viability but can also effectively inhibit the cholesterol efflux induced by AIBP + APOA-I (Additional file 1: Figure S3). Further analysis showed that CsA treatment also abolished the inhibitory effect of AIBP + APOA-I on cell migration and invasion and tumor-induced angiogenesis (Fig. 6c–f).

Collectively, our data indicate that the anti-tumor effect of AIBP and APOA-I in combination is mainly dependent on promoting the cholesterol efflux.

## Discussion

Recently, disruption of lipid rafts of malignant cells has been considered an essential strategy for the prevention and treatment of cancer [6]. In this study, we found that low expression levels of AIBP and APOA-I are associated with the degree of malignancy in intestinal tumors. Combined with AIBP and APOA-I inhibited tumor growth and metastasis as well as cell migration, invasion and tumor-induced angiogenesis. Mechanically, cholesterol efflux promoted by AIBP + APOA-I interfered with the raft-related signaling pathway.

Prior studies have shown that AIBP often cooperates with APOA-I to regulate the RCT pathway in various types of cells. However, the expression patterns of AIBP and APOA-I in malignant tissues remain poorly understood. In this study, we found that both AIBP expression and APOA-I expression decreased gradually with progression from an adenoma to an advanced tumor. In this process, the decreasing rates of AIBP and APOA-I levels are not entirely synchronized. For example, in low-malignancy adenoma or colon cancer cells, the expression of one of these proteins was always significantly higher or lower than that of the other. However, in advanced tumors or high-malignancy colon cancer cells, the expression of both proteins was consistently decreased to a minimum. These results suggested that simultaneous low expression levels of both proteins likely represent a critical step in the malignant transformation of tumor cells. Therefore, the expression levels of AIBP and APOA-I can be used as an indicator of malignancy of intestinal cancer.

APOA-I was reported to have anti-tumor activity in some xenograft tumor models [10, 36], but its therapeutic effect is limited in the inherited tumor model [37]. Consistently, we found that APOA-I treatment alone can inhibit colon cancer cell-mediated tumor growth to a certain extent but has no apparent inhibitory effect on tumor growth in Apc^min/+^ mice. However, the addition of AIBP enhanced the anti-tumor effect of APOA-I both in xenograft and inherited tumor models. Similar to in vitro studies, APOA-I treatment alone can induce cholesterol efflux to a certain degree but was insufficient to produce significant changes in cellular behavior due to a lower rate of cholesterol efflux. In contrast, AIBP hardly promoted cholesterol efflux when APOA-I was absent due to the lack of a cholesterol acceptor. Thus, APOA-I is necessary for cell cholesterol efflux, whereas AIBP serves as an accelerator to augment this effect triggered by APOA-I. These results suggested that the combined anti-tumor effect is closely related to enhanced cholesterol efflux. Whether AIBP in combination with APOA-I promotes other anti-tumor pathways, such as anti-inflammatory or anti-oxidation pathways, requires further study.

Cell migration and invasion are essential processes for cancer metastasis. Assembly of the integrin-based adhesion structure is critical for effective cell movement. Recent evidence suggests that integrin β1 clustering and functioning are regulated by membrane rafts, which provide a large platform for assembling different proteins to facilitate the migration-related signaling pathway [31]. Moreover, MT1-MMP, as one of the raft-affiliated matrix metalloproteinases (MMPs), has been shown to contribute to the invasive abilities of tumor cells by activating pro-MMP-2 [32, 38, 39]. In the present study, AIBP combined with APOA-I promoted cholesterol efflux from the membrane and directly affected the tight packing and stabilization of lipid rafts, thus impairing proper localization of integrin β1 and MT1-MMP and eventually migration- and invasion-related signaling cascades. On the other hand, the combination of AIBP and APOA-I inhibited tumor-induced angiogenesis, which is an essential process for tumor cell growth and metastasis. Therefore, the combination of AIBP and APOA-I not only restricts cancer cell migration and invasion but also blocks hematogenous metastasis.

APOA-I-dependent cholesterol efflux involves a Ca^2+^-dependent endocytic pathway, followed by recycling and subsequent release of the nascent lipoprotein particle from the cell [40, 41]. Therefore, CsA acts as a potent inhibitor of cholesterol efflux by suppressing the internalization of APOA-I [42, 43]. Here, CsA treatment compromised the inhibitory role of AIBP combined with APOA-I in colon cancer cell-mediated tumor growth as well as cell migration, invasion and tumor-induced angiogenesis. These results suggested that the anti-tumor activity of AIBP combined with APOA-I is dependent on cholesterol efflux.

## Conclusions

In this study, we found that the combination of AIBP and APOA-I exerts a significant inhibitory effect on intestinal tumor growth and liver metastasis. This effect was achieved by promoting cholesterol efflux and subsequent suppression of the lipid raft-associated signaling pathway. Taken together, these findings suggest that AIBP and APOA-I in combination serve as a natural cholesterol-depleting agent and could be of therapeutic value in preventing metastasis of human CRC and potentially other cancers.

## Additional files

**Additional file 1.** **Figure S1.** Stable overexpression of AIBP and/or APOA-I in HCT116 and SW620 cell lines. **Figure S2.** ELISA assay for serum APOA-I and AIBP levels in Apc ^Min/+^ mice**. Figure S3.** Effects of CsA at varying concentrations on cholesterol efflux and the viability of colon cancer cells.

**Additional file 2.** **Table S1.** The information of the cell lines. **Table S2.** QPCR primer list. **Table S3.** Associations of AIBP and APOA-I expression with clinico-pathological factors of the patients with colon cancer. **Table S4.** Statistical data of xenografted tumor volume and weight. **Table S5.** Hazard’s Ratio.

## Data Availability

The datasets used and/or analysed during the current study are available from the corresponding author on reasonable request.

## Abbreviations

AIBP: APOA-I binding protein
MβCD: methyl-β-cyclodextrin
MT1-MMP: membrane type I matrix metalloproteinase
CAV-1: caveolin-1
