# Supplementary figures and images for: AIBP and APOA-I synergistically inhibit intestinal tumor growth and metastasis by promoting cholesterol efflux
Source: J Transl Med. 2019 May 17;17:161. doi: 10.1186/s12967-019-1910-7 (PMC6524272; doi:10.1186/s12967-019-1910-7)

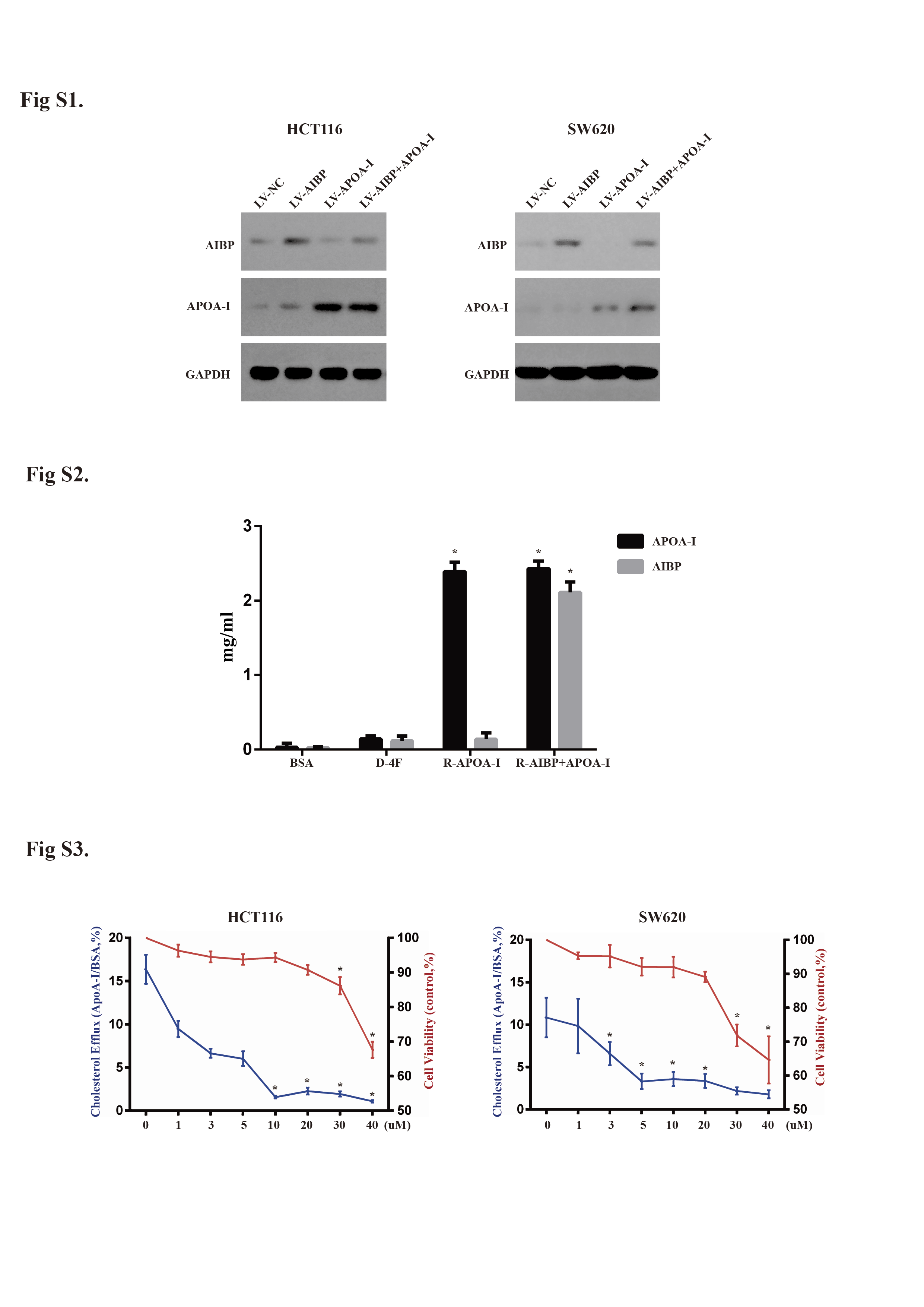

Supplement: Supplementary file 1 — Additional file 1. Figure S1. Stable overexpression of AIBP and/or APOA-I in HCT116 and SW620 cell lines. Figure S2. ELISA assay for serum APOA-I and AIBP levels in Apc Min/+ mice. Figure S3. Effects of CsA at varying concentrations on cholesterol efflux and the viability of colon cancer cells. [file 12967_2019_1910_MOESM1_ESM.tif]
