# Supplementary material for: AIBP and APOA-I synergistically inhibit intestinal tumor growth and metastasis by promoting cholesterol efflux
Source: J Transl Med. 2019 May 17;17:161. doi: 10.1186/s12967-019-1910-7 (PMC6524272; doi:10.1186/s12967-019-1910-7)
Supplement: Supplementary file 2 — Additional file 2. Table S1. The information of the cell lines. Table S2. QPCR primer list. Table S3. Associations of AIBP and APOA-I expression with clinico-pathological factors of the patients with colon cancer. Table S4. Statistical data of xenografted tumor volume and weight. Table S5. Hazard’s Ratio. [file 12967_2019_1910_MOESM2_ESM.docx]

**Table S1. The Information of The Cell Lines**

| **Name of Cell Lines** | **Species** | **Anatomical Location** | **Cancer or Normal** |
| --- | --- | --- | --- |
| HEK293 | ***Homo sapiens*** | Renal Epithelial Cells | Normal |
| HIEC | ***Homo sapiens*** | Intestinal Epithelial Cells | Normal |
| 841 | ***Homo sapiens*** | Colon Epithelial cells | Normal |
| Caco-2 | ***Homo sapiens*** | Colon Epithelial cells | Colorectal Adenocarcinoma |
| SW480 | ***Homo sapiens*** | Colon Epithelial cells | Colorectal Adenocarcinoma |
| HT29 | ***Homo sapiens*** | Colon Epithelial cells | Colorectal Adenocarcinoma |
| LS174-T | ***Homo sapiens*** | Colon Epithelial cells | Colorectal Adenocarcinoma |
| RKO | ***Homo sapiens*** | Colon Epithelial cells | Colorectal Carcinoma |
| LOVO | ***Homo sapiens*** | Colon Epithelial cells | Colorectal Adenocarcinoma |
| SW620 | ***Homo sapiens*** | Colon Epithelial cells | Colorectal Carcinoma |
| HCT116 | ***Homo sapiens*** | Colon Epithelial cells | Colorectal Carcinoma |
| HUVECs | ***Homo sapiens*** | Human Umbilical Vein Endothelial Cells | Normal |

**Table S2. QPCR Primer List**

|  | **Forward (5'to 3')** | **Reverse (5'to 3')** |
| --- | --- | --- |
| human-AIBP-QPCR primer | GCGTGGACCAACTTATGGAAC | GGCTCGTAGCCAAAGAGTTTG |
| human-APOA-I-QPCR primer | GTGACCTCCACCTTCAGCAA | CCTCCTGCCACTTCTTCTGG |
| human-GAPDH-QPCR primer | TGGCACCGTCAAGGCTGAGAA | TGGTGAAGACGCCAGTGGACTC |
| mouse-AIBP-QPCR primer | ATTGCCAAGGCTTATCCCCC | CCAGTGAAGAGGGGCTTGTT |
| mouse-APOA-I-QPCR primer | TTAGTCAGCTGCAGGAACGG | GTAGGGCTGCACCTTCTGTT |
| mouse-beta actin-QPCR primer | GGCTGTATTCCCCTCCATCG | CCAGTTGGTAACAATGCCATGT |

**Table S3. Association of AIBP and APOA-I expression with Clinic-pathological factors from patients with colon cancer**

| **Clinicopathological variables** | **N** | **AIBP expression score (means±SD)** | | **P value** | **APOA-I expression score (means±SD)** | | **P value** |
| --- | --- | --- | --- | --- | --- | --- | --- |
|  |  |  |  |  |  |  |  |
| Age (years) |  |  |  |  |  |  |  |
| < 60 | 29 | 5.25 ± 1.70 | | 0.0025 | 4.67 ± 1.51 | | 0.77 |
| ≥60 | 19 | 4.06 ± 1.82 | |  | 4.82 ± 1.97 | |  |
| Tumor differentiation |  |  | |  |  | |  |
| Well | 11 | 5.22 ± 1.66 | |  | 5.88 ± 1.01 | |  |
| Moderate | 16 | 4.98 ± 1.35 | |  | 4.86 ± 1.75 | |  |
| Poor | 20 | 2.75 ± 1.26 | | < 0.01 | 3.86 ± 1.82 | | < 0.05 |
| Infiltration depth |  |  |  |  |  |  |  |
| T1+T2 | 15 | 2.77 ± 1.74 | | 0.29 | 5.71 ± 1.34 | | < 0.05 |
| T3+T4 | 32 | 4.35 ± 1.23 | |  | 4.32 ± 1.82 | |  |
| Lymph node metastasis |  |  |  |  |  |  |  |
| N0 | 21 | 5.56 ± 3.17 | | 0.34 | 4.83 ± 1.82 | | 0.8 |
| N1-3 | 26 | 3.47 ± 1.73 | |  | 4.70 ± 1.78 | |  |
| Distant metastasis |  |  |  |  |  |  |  |
| M0 | 20 | 3.84 ± 1.38 | | 0.52 | 4.93 ± 1.82 | | 0.59 |
| M1 | 27 | 4.34 ± 1.41 | |  | 4.64 ± 1.78 | |  |
| TNM stage |  |  |  |  |  |  |  |
| Ⅰ | 14 | 6.22 ± 3.45 | |  | 5.96 ± 0.96 | |  |
| Ⅱ | 13 | 3.45 ± 1.56 | | < 0.05 | 4.86 ± 1.75 | | < 0.05 |
| Ⅲ | 20 | 2.19 ± 1.21 | | < 0.01 | 3.31 ± 1.53 | | < 0.01 |

**Table S4. Statistical Data of Xenografted Tumors Volume and Weight**

**a.**

| Cell line | Overexpression | Tumour Volume (mm^3^) | | | | Tumour Weight (g) | | | |
| --- | --- | --- | --- | --- | --- | --- | --- | --- | --- |
|  |  | Mean | N | SD | P value | mean | n | SD | P value |
| HCT116 | LV-NC | 311.74 | 5 | 88.07 | <0.01 | 0.804 | 5 | 0.102 | <0.01 |
|  | LV-AIBP | 248.87 | 5 | 72.71 | <0.01 | 0.700 | 5 | 0.071 | <0.01 |
|  | LV-APOA-I | 70.63 | 5 | 18.59 | <0.01 | 0.518 | 5 | 0.051 | <0.05 |
|  | LV-AIBP+APOA-I | 27.72 | 5 | 13.87 |  | 0.382 | 5 | 0.094 |  |
| SW620 | LV-NC | 315.25 | 5 | 113.44 | <0.01 | 0.808 | 5 | 0.102 | <0.01 |
|  | LV-AIBP | 318.60 | 5 | 55.89 | <0.01 | 0.796 | 5 | 0.040 | <0.01 |
|  | LV-APOA-I | 215.75 | 5 | 65.93 | <0.01 | 0.708 | 5 | 0.083 | <0.01 |
|  | LV-AIBP+APOA-I | 55.85 | 5 | 48.04 |  | 0.458 | 5 | 0.073 |  |

**b.**

| Tumour Volume (mm^3^) | | | | | | | | | | | | | |
| --- | --- | --- | --- | --- | --- | --- | --- | --- | --- | --- | --- | --- | --- |
| Days | LV-NC （Means±SD） | | | LV-AIBP （Means±SD） | | | LV-NC+InJ-APOA-I（Means±SD） | | | LV-AIBP+InJ-APOA-I（Means±SD） | | | P value |
| 0 | 0.00 | ± | 0.00 | 0.00 | ± | 0.00 | 0.00 | ± | 0.00 | 0.00 | ± | 0.00 |  |
| 3 | 2.73 | ± | 1.37 | 2.13 | ± | 0.57 | 0.90 | ± | 0.00 | 1.30 | ± | 0.57 |  |
| 6 | 17.27 | ± | 2.66 | 13.05 | ± | 1.23 | 12.47 | ± | 2.31 | 10.40 | ± | 3.56 |  |
| 9 | 56.67 | ± | 25.32 | 42.88 | ± | 11.50 | 33.83 | ± | 13.80 | 23.13 | ± | 9.03 |  |
| 12 | 127.90 | ± | 36.49 | 115.90 | ± | 24.85 | 98.70 | ± | 4.81 | 53.63 | ± | 8.71 |  |
| 15 | 276.50 | ± | 39.88 | 229.55 | ± | 76.42 | 175.10 | ± | 18.55 | 113.80 | ± | 16.55 |  |
| 18 | 349.10 | ± | 22.91 | 282.60 | ± | 67.32 | 212.37 | ± | 14.05 | 146.33 | ± | 12.92 | <0.01 |
| 21 | 600.23 | ± | 16.31 | 495.10 | ± | 46.65 | 383.03 | ± | 41.57 | 196.23 | ± | 23.66 | <0.01 |

**c.**

| Overexpression | Tumour Weight (g) | | | |
| --- | --- | --- | --- | --- |
|  | Means±SD | | | P value |
| LV-NC | 0.75 | ± | 0.10 |  |
| LV-AIBP | 0.84 | ± | 0.06 |  |
| R-APOA-I | 0.54 | ± | 0.03 | <0.05 |
| LV-AIBP+R-APOA-I | 0.33 | ± | 0.09 | <0.05 |

**Table S5.** **Hazard's Ratio**

| Parameters | LV-AIBP+APOA-I VS.  LV-NC | LV-AIBP+APOA-I  VS.  LV-AIBP | LV-AIBP+APOA-I  VS.  LV-APOA-I | LV-AIBP  VS.  LV-NC | LV-NC  VS. LV-NC |
| --- | --- | --- | --- | --- | --- |
| Hazard Ratio (logrank) | 0.2446 | 0.3635 | 0.4259 | 0.4749 | 0.441 |
| 95% CI | 0.017 ~ 0.303 | 0.067 ~ 0.619 | 0.104 ~ 0.883 | 0.110 ~ 1.184 | 0.088 ~ 0.990 |
| P value | 0.0015 | 0.0149 | 0.0497 | 0.1234 | 0.0792 |
